# Biomechanical compensatory chain and residual compensation potential in aging spinal deformity: a narrative review and multimodal assessment framework

**DOI:** 10.3389/fmed.2026.1801298

**Published:** 2026-03-27

**Authors:** Yingqi Geng, Ronghui Cai, Runhui Zhang, Yuqing Sun

**Affiliations:** Department of Spine Surgery, Capital Medical University Affiliated Beijing Jishuitan Hospital, Beijing, China

**Keywords:** adult spinal deformity, biomechanics, compensatory chain, residual compensation potential, sagittal imbalance, secondary degeneration, the elderly

## Abstract

**Background/objectives:**

As a prevalent condition among the elderly, adult spinal deformity (ASD) initiates progressive decompensation not only in the spine but also throughout the lower limbs. This systemic impairment reduces the elderly’s mobility, elevates the risk of falls, and consequently places a considerable burden on public health. Current assessment approaches frequently neglect the physiological ‘cost’ of such compensatory adaptations. This review aims to clarify the biomechanical relationship between sagittal spinal imbalance and secondary lower limb pathologies, and to propose “Residual Compensation Potential” (RCP) innovatively as a quantifiable framework for evaluation.

**Methods:**

A narrative review was conducted by retrieving PubMed, Web of Science, and Embase databases (up to December 2025). We synthesized evidence regarding spinal-pelvic parameters, lower limb kinematics, and joint degeneration mechanisms to construct a ‘imbalance-compensation-degeneration’ closed-loop model.

**Results:**

The compensatory chain follows a hierarchical sequence: posterior pelvic tilt, hip hyperextension, knee flexion, and ankle dorsiflexion. While initially protective, prolonged maintenance of these postures alters mechanical loading and leads to specific degenerative changes: anterior hip cartilage wear from hyperextension, patellofemoral overload from knee flexion, and Achilles tendinopathy from sustained dorsiflexion. We introduce the RCP as a proposed framework, which combines EOS imaging, 3D gait analysis, and surface electromyography (sEMG) to quantify the remaining compensatory capacity of these joints. The resulting RCP score stratifies patients into compensation-preserved or compensation-depleted categories, informing targeted surgical and rehabilitative planning.

**Conclusion:**

The evaluation of adult spinal deformity should extend beyond static spinal alignment to include the functional status of the lower limbs. The proposed RCP framework offers a novel method to assess the risk of secondary joint degeneration and postoperative imbalance. Consequently, clinical management must evolve from a focus on radiographic correction alone toward the holistic recovery of the entire compensatory chain, ensuring that spinal realignment translates into stable and functional gait.

## 1 Introduction

Adult spinal deformity (ASD) refers to a three-dimensional structural abnormality of the spine developing after skeletal maturity, predominantly affecting the lumbar and thoracolumbar regions. Its causes include iatrogenic deformities, traumatic deformities and degenerative deformities resulting from intervertebral disc disease, etc. (1, 2). With aging global populations, the incidence and prevalence of ASD caused by degenerative factors are rising (3, 4). It is reported that the prevalence of spinal deformity exceeds 32–68% among the aging population, and increases significantly with age. By 2050, it is projected that over 60 million older adults in the United States will be affected by some form of spinal deformity (5, 6). These patients frequently suffer from refractory musculoskeletal pain, progressive postural imbalance, and neurological dysfunction, resulting in profound limitations in activities of daily living, markedly reduced health-related quality of life (HRQoL), heightened risk of falls and fractures, accelerated frailty, and progressive loss of independence-ultimately imposing a substantial burden on patients, caregivers, and healthcare systems (7, 8).

Although ASD encompasses both coronal and sagittal malalignment, research has increasingly concentrated on sagittal plane imbalance due to its closer links to clinical symptoms and HRQoL (9). In response to sagittal imbalance, the body recruits pelvic and lower limb compensations, such as increased pelvic tilt and knee flexion, to preserve upright stance. The sustained maintenance of this compensated posture promotes accelerated wear and degeneration, culminating in secondary arthropathy (10). Although the ‘spine-to-joint’ concept has been introduced, current evidence predominantly focuses on the impact of joints on the spine (11). Evidence is scattered across disciplines from spinal surgery to biomechanics, without a clear framework to systematically link lower-limb compensatory mechanics to secondary joint degeneration in sagittal deformity.

**Figure 1:**
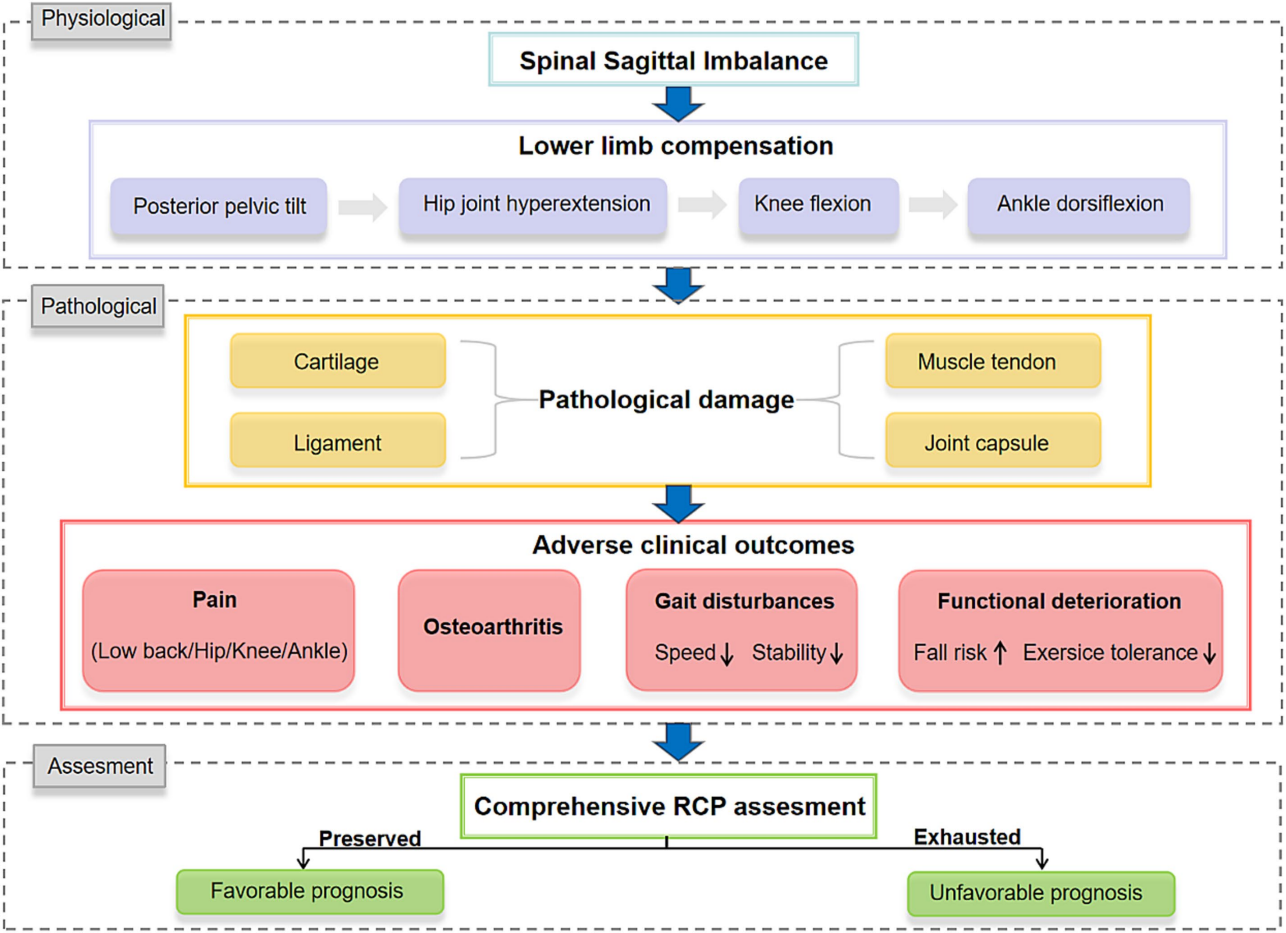
The cascade of lower extremity compensation and secondary degeneration in spinal sagittal imbalance: from mechanism to clinical implication.

This narrative review synthesizes the biomechanical compensatory chain in geriatric ASD and introduces Residual Compensation Potential (RCP) as a proposed multimodal framework to address the current disconnect between radiographic correction and functional outcomes. Figure 1 illustrates the overall framework of lower-limb compensation and secondary degeneration in spinal sagittal imbalance (Figure 1). Future research should explore the molecular mechanisms linking abnormal mechanical loading to local inflammation, as well as the mediating role of mechanobiological signals, to inform targeted therapeutic strategies.

## 2 Method

We conducted a comprehensive literature search in PubMed, Web of Science, and Embase up to December 2025 using keywords related to adult spinal deformity, lower limb compensation, and biomechanics. The inclusion criteria were studies reporting the link between specific lower-limb compensatory strategies and secondary degeneration of the lower limbs or the application of quantitative assessment techniques to the above compensation or degeneration processes. Exclusion criteria included non-English articles, and studies without a description of spinal deformity. Crucially, to ensure that the observed lower limb mechanics were indeed compensatory responses to spinal malalignment rather than manifestations of primary joint pathology, we also excluded studies that included participants with congenital or primary lower limb deformities. Data extraction was performed independently by two authors, with disagreements resolved through discussion. Priority was given to studies integrating radiographic parameters with gait analysis or joint degeneration.

## 3 Pathological and biomechanical basis of sagittal imbalance of the spine

### 3.1 Normal sagittal alignment and key parameters

The human spine exhibits characteristic sagittal curvatures—cervical lordosis (CL), thoracic kyphosis (TK), and lumbar lordosis (LL)—that alternate in direction. This configuration is fundamental to bipedal stability, efficiently transmitting body weight to the ground while maintaining an upright posture (12). Biomechanically, the spine and pelvis form a closely coupled unit, with their harmonious interplay essential for global postural balance (13). A key anatomical determinant is the Pelvic Incidence (PI), an individual-specific angle reflecting inherent pelvic morphology. It is defined by the angle between the line perpendicular to the sacral plate at its midpoint and the line connecting this point to the bicoxofemoral axis. Two related positional parameters are Pelvic Tilt (PT), the angle between the sacral-femoral line and the vertical, and Sacral Slope (SS), the angle between the sacral plate and horizontal. These three parameters are intrinsically linked by the equation: PI=PT+SS (14–17).

Normally, the C7 plumb line (C7PL) passes through the posterosuperior corner of the sacral endplate, positioning the center of gravity directly above the hip joints and maintaining the sagittal vertical axis (SVA) within ±50 mm. Anterior displacement of the C7PL and a significant increase in SVA indicate a state of positive sagittal imbalance. Clinically, SVA, the PI–LL mismatch, and PT are widely used to evaluate spinopelvic alignment. Among these, the PI–LL mismatch reflects the harmony between lumbar lordosis and pelvic morphology. It serves as a key indicator for assessing sagittal spinal balance (18, 19). A PI–LL value >10° suggests insufficient lumbar lordosis or compensatory pelvic retroversion, whereas a value >20° is typically associated with marked postural imbalance and compensatory exhaustion (20).

### 3.2 The formation and progression of degenerative changes

Degenerative spinal deformity progresses from localized structural degeneration to global imbalance, characterized primarily by loss of LL, increased TK, and anterior shifting of the body’s center of gravity. With aging and sustained mechanical loading, degenerative changes in intervertebral discs, facet joints, ligaments, and vertebral bone collectively contribute to the loss of sagittal alignment (21).

Intervertebral disc degeneration often constitutes the initial stage of spinal imbalance. Declining function of nucleus pulposus cells and loss of extracellular matrix components reduce proteoglycan and water content within the disc, leading to disc space collapse and diminished shock-absorbing capacity. Consequently, the anterior column undergoes compression while the posterior column is subjected to tension, resulting in a decrease of lumbar lordosis (22, 23). On MRI, common manifestations include diminished T2 signal intensity and disc space narrowing, with the severity of degeneration commonly graded according to the Pfirrmann classification (24). Following loss of disc height, increased loading on the facet joints induces prolonged abnormal stress, which promotes cartilage wear, osteophyte formation, and hypermobility of the articular processes (25). Simultaneously, compensatory hypertrophy of the ligamentum flavum, along with laxity or ossification of the posterior longitudinal ligament, further compromises posterior spinal stability (26, 27). These changes facilitate the progression from focal kyphosis to global sagittal imbalance.

Osteoporotic vertebral compression fractures significantly contribute to spinal imbalance. Anterior vertebral collapse results in wedging deformity, which increases TK and establishes a vicious cycle of “compression–deformation–recompression.” Studies indicate that the degree of vertebral wedging is positively correlated with the spinal kyphotic angle and can progressively amplify sagittal malalignment, thereby altering the overall mechanical load transmission pathway (28, 29).

The progression of degenerative spinal deformity follows a sequential process characterized by local degeneration, global imbalance, and eventual failure of compensation. The synergistic degeneration of intervertebral discs, facet joints, ligaments, and vertebral bone forms the biomechanical basis of sagittal imbalance, which is further exacerbated by osteoporosis. Understanding this pathomechanism facilitates early identification of high-risk patients and supports the optimization of clinical intervention strategies.

### 3.3 The overall biomechanical consequences of imbalance

In patients with ASD, sagittal imbalance may disrupt the energy-efficient postural equilibrium of body. According to Dubousset’s “cone of economy” theory, the center of gravity should remain within a stable region that minimizes energy expenditure during upright posture. Once it shifts beyond this zone, compensatory activation of paraspinal muscles is required to maintain balance (30). Sustained compensatory effort leads to muscle fatigue, lactic acid accumulation, chronic pain, and eventually atrophy and fatty infiltration, thereby impairing dynamic stability and perpetuating a vicious cycle of imbalance–compensation–deterioration (31, 32). In cohort studies, a SVA exceeding 50 mm is associated with increased fall risk, higher daily energy expenditure, and significantly reduced quality of life (33, 34).

To evaluate spinal imbalance and guide treatment, the International Society for the Study of the Lumbar Spine (ISSLS) introduced the SRS-Schwab classification. This system utilizes the PI–LL mismatch, SVA, and PT as key parameters to assess spinopelvic sagittal alignment. A PI–LL difference ≤ 10° is considered normal, 10°–20° indicates moderate imbalance, and > 20° represents severe imbalance. Furthermore, an SVA > 95 mm or PT > 30° suggests that compensatory mechanisms are nearing exhaustion. Table 1 summarizes the normal ranges, abnormal thresholds, grading, and clinical relevance of these parameters (Table 1).

**Table 1:** SRS-Schwab classification criteria for sagittal parameters.

Parameter	Normal range	Grade	Clinical significance
PI-LL (°)	≤10°	>10°: Moderate (Grade+) >20°: Marked (Grade++)	Evaluating the relationship between the pelvis and the lumbar lordosis
SVA (mm)	≤50 mm	>50 mm: Moderate (Grade+) >95 mm: Marked (Grade++)	Assessing global spinal alignment and evaluating the restoration of the patient after surgical intervention
PT (°)	≤20°	>20°: Moderate (Grade+) >30°: Marked (Grade++)	Reflecting the compensatory status of the pelvis in spinal deformity

Collectively, sagittal imbalance triggers a compensatory lower-limb kinetic chain involving the pelvis, hip, knee, and ankle joints to maintain equilibrium. The specific mechanisms and regulatory processes involved will be detailed in the following section.

## 4 Biomechanical remodeling of the lower limbs

### 4.1 Overall pattern of lower limb chain compensation

This compensatory process of lower limbs exhibits clear hierarchical and temporal characteristics: ① Core-level compensation: When SVA or PI–LL exceeds abnormal thresholds (e.g., Table 1), the pelvis rotates posteriorly to shift the line of gravity backward and the hip is forced into hyperextension to maintain upright balance. ② Secondary-level compensation: When proximal compensation is exhausted, knee flexion and ankle dorsiflexion are simultaneously recruited to maintain the projection of the center of gravity within the base of support (35). Table 2 summarizes the corresponding staging of lower-limb compensatory status (Table 2).

**Table 2:** Characteristics of each phase of lower limb compensation.

Phase	Risky joints	Compensatory manifestations	Main symptoms
I (Pelvis/Hip Dominated)	Hip	Pelvic retroversion/Hip hyperextension/Increased pelvic sway during gait	Fatigue or aching in lower back and hips, aggravated by prolonged standing
II (Knee/Ankle Involvement)	Knee /Ankle	Knee flexion/Increased ankle dorsiflexion/Weakened push-off during gait	Anterior knee pain, ankle discomfort and limited walking distance
III (Decompensation Phase)	Whole lower-limb joints	Overall postural instability and markedly abnormal gait	Migratory multi-joint pain, difficulty in standing and walking

Musculoskeletal modeling (Lafage et al.) suggests that when the compensatory effects of the ankle, knee, and hip joints are progressively removed, the SVA increases from 65 mm in the compensated state to 95 mm and 120 mm (36). This may indicate the importance of lower limb compensation in maintaining overall upper body balance. Diebo et al. further pointed out that as PI-LL increases, thoracic and pelvic compensation becomes exhausted, and the compensatory role of the knee and ankle joints will become more pronounced (37).

Notably, lower-limb chain compensation is not merely a passive response to spinal sagittal imbalance, but also an active strategy employed by the body to achieve overall sagittal alignment. For clinicians, identifying the sequence and threshold limits of this compensatory chain is essential to evaluate the residual compensatory potential and to determine the appropriate extent of surgical correction.

### 4.2 Pelvis and hip joint: core compensation link

**Figure 2:**
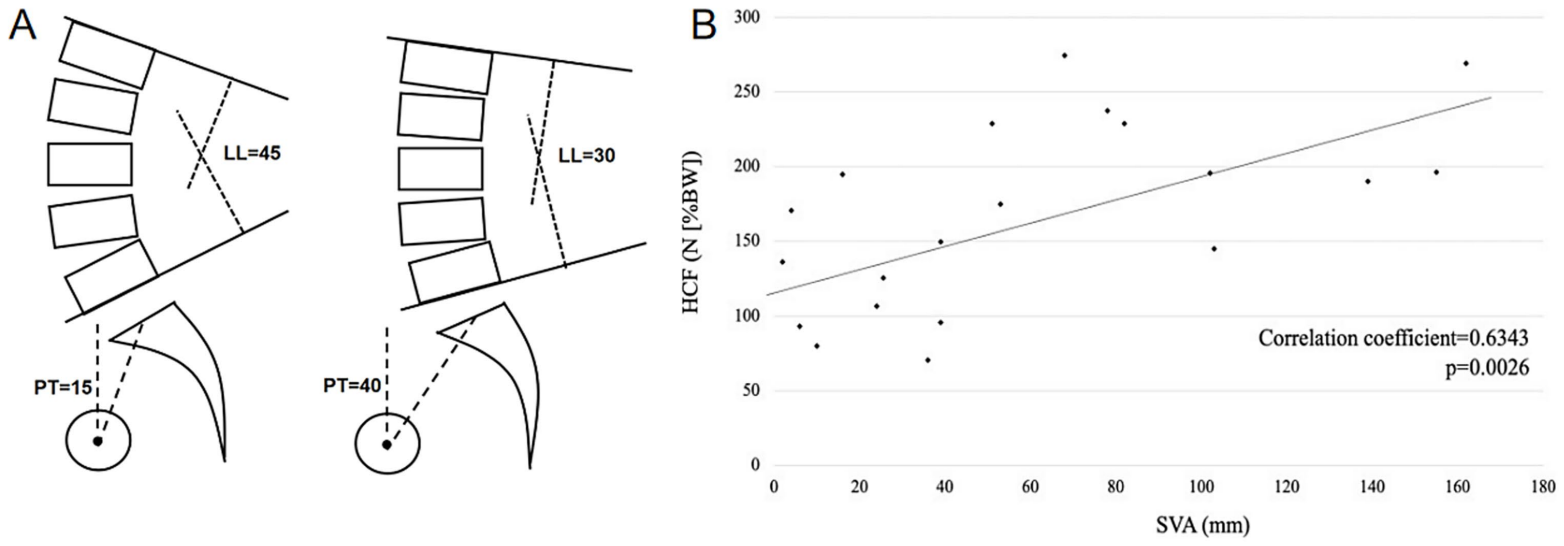
The pelvic pattern diagram and HCF linear diagram of sagittal imbalance patient. **(A)** The changes in pelvic parameters and lumbar lordosis before and after spinal sagittal imbalance. **(B)** The linear relationship between hip contact force and SVA. Adapted with permission40. Copyright 2021, association between global sagittal malalignment and increasing hip joint contact force, analyzed by a novel musculoskeletal modeling system.

Posterior pelvic tilt serves as the initial compensatory mechanism in sagittal spinal imbalance. It accommodates lower lumbar lordosis by altering the orientation of the sacral slope, while simultaneously shifting the trunk’s center of gravity posteriorly through pelvic rotation. In a clinical cohort (Wang et al.), SVA demonstrated a significant linear correlation with PT (*p* < 0.01). Moreover, for every 10° increase in PI-LL, the PT/PI ratio increased synchronously by 0.09–0.10. This may suggest a central role of pelvic compensation in restoring sagittal spinal alignment. This posterior rotation plays a functional role in rebalancing energy expenditure, thereby serving as a primary defense of upright posture. Figure 2A illustrates changes in pelvic retroversion and related parameters before and after the development of sagittal imbalance (38, 39).

When pelvic retroversion reaches its maximum limit, the hip joints engage in secondary compensation through hyperextension. This involves sustained high-intensity contraction of the hip extensors (gluteus maximus, adductor magnus) and passive stretching of the hip flexors (iliopsoas, rectus femoris), leading to elevated anterior soft-tissue tension and abnormal joint loading. In a musculoskeletal modeling study (Miura et al.), patients with a SVA > 50 mm exhibited a 75.2% increase in hip contact force (HCF) compared to those with normal alignment. This may suggest a marked rise in energy expenditure and degenerative risk due to compensatory overloading. Furthermore, hip flexion torque in the high-SVA group increased from 0.1 Nm to 2.9 Nm. This indicate that anterior structures such as the joint capsule and iliofemoral ligament are subjected to abnormal tensile forces (40, 41). Chronic exposure to this high-load state appears to significantly elevate the risk of anterior cartilage wear, marginal osteophyte formation, and secondary osteoarthritis. The linear relationship between SVA and HCF is illustrated in Figure 2B.

The pelvis and hip joint function in synergy to maintain sagittal balance: pelvic retroversion offers angular compensation, while hip extension provides a stable pivot. These two mechanisms are interdependent; limitations in hip extension or the presence of hip flexor contracture reduce pelvic retroversion capacity, thereby increasing SVA. Clinically, this explains why some patients remain in a forward-leaning posture following spinal deformity surgery—often not due to inadequate spinal correction, but rather exhaustion of the hip–pelvic compensatory reserve. Thus, surgical planning and rehabilitation assessment should include concurrent measurement of PT, SS, hip extension angle, and HCF as key indicators of sagittal balance recovery potential. As the central hub of the lower limb compensatory chain, failure of the pelvic–hip segment shifts compensatory demands distally to the knee and ankle. This will be expanded upon in the following section (41, 42).

### 4.3 Knee joint and ankle joint: secondary compensatory links

When pelvic retroversion capacity is exhausted and hip extension is limited, compensatory mechanisms progress further along the kinetic chain to the knee and ankle. At this stage, the knee commonly exhibits mild flexion during stance. In a cohort study of healthy young adults (Lee et al.), changing the knee from full extension to 30° flexion reduced LL from 50.7° to 42.7° and increased the SVA from −1.2 mm to 47.6 mm, without significant alterations in PT or SS (43). These findings suggest that knee flexion can influence sagittal balance independent of pelvic rotation. A similar compensatory pattern is observed under pathological conditions; a systematic review of gait analysis in patients with sagittal imbalance indicates that knee flexion during gait may serve as a common compensatory strategy to shift the center of gravity posteriorly (44).

Building on knee flexion, ankle dorsiflexion further supports lower limb stability. A Meta-analysis indicate that in patients with ASD, ankle plantarflexion range of motion (ROM) is notably reduced during mid-stance and propulsion, while dorsiflexion increases markedly in the late stance phase (44). This elevated dorsiflexion may help prevent tripping by improving foot clearance and moving the center of gravity posteriorly. However, it appears to compromise forward propulsion efficiency and elevate energy expenditure. Collectively, although ankle dorsiflexion likely aids balance maintenance, it may do so at the expense of higher metabolic cost and possible muscle fatigue.

The compensatory mechanisms of the knee and ankle joints can partially restore sagittal plane balance in the short term, but long-term maintenance will significantly increase the risk of joint disease. This provides a mechanical basis for the subsequent discussion on ‘lower limb function and energy cost’ (45, 46).

### 4.4 Functional and clinical consequences of changes in lower limb biomechanics

While compensatory lower-limb mechanisms sustain postural balance in the short term, they entail substantial long-term consequences for muscles, joints, and overall energy metabolism.

The primary physiological outcome of serial compensatory mechanisms is the sustained overload of lower-limb extensor muscles, including the gluteus maximus (GM), biceps femoris (BF), and thoracolumbar erector spinae (TES). Surface electromyography study (Banno et al.) suggests elevated muscle activity in the GM, BF, and TES in patients with ASD compared to controls. Concurrently, low-frequency sustained discharge in the lumbar erector spinae appears to reflect a high-energy state necessary for maintaining compensation (47). Although this prolonged muscle overactivation may support short-term postural balance, it likely accelerates muscular metabolism and fatigue, which may contribute to the characteristic lumbar and lower-limb fatigue and pain reported in this population. Furthermore, a systematic review reported an increase in average oxygen consumption (VO₂) from 9.9 to 13.8 mL·kg^−1^·min^−1^ in individuals with spinal deformity, suggesting a substantially elevated energy cost during standing and walking in ASD patients compared to healthy individuals (48).

At the joint level, sustained knee flexion and hip hyperextension contribute to altered torque transmission. A mathematical modeling study (Takabayashi et al.) demonstrates that when the knee joint extension torque reaches its maximum value (240 Nm), the patellofemoral joint stress reaches its maximum value (22.4 N/mm (2)) at 10° of knee flexion. This redistribution of mechanical load may accelerate articular cartilage wear. Additionally, chronic stretching of the gastrocnemius can elevate Achilles tendon tension, creating a zone of stress accumulation in the distal lower limb. Clinically, approximately 63% of patients with long-term compensatory patterns present with lower-limb joint pain, which appears to reflect the progressive pathological nature of these adaptations (49).

Lower limb compensation also impacts overall locomotor efficiency. In a three-dimensional gait analysis (Kawkabani et al.), patients with ASD demonstrated a reduced walking speed of approximately 0.2 m/s, a decrease in step frequency by 14 steps per minute, and a prolonged double support phase by 0.08 s compared with healthy controls (50). These alterations may reflect a compensatory state that contributes to diminished propulsive force and increased energy expenditure. Monitoring of metabolic equivalents (METs) further indicated that energy consumption during walking was about 30% higher in patients than in controls at matched speeds, suggesting that compensatory gait patterns may enhance stability at the cost of reduced efficiency (48). Additionally, excessive ankle dorsiflexion and an anterior shift in plantar pressure appear to promote gait instability, potentially elevating fall risk.

Collectively, while lower limb compensation helps maintain sagittal balance in the short term, it imposes a high long-term metabolic demand on muscles, increases joint stress, and reduces walking efficiency. This biomechanical state—characterized by high energy consumption and low efficiency—not only diminishes quality of life but also predisposes individuals to degenerative joint changes and chronic pain syndromes. Understanding these functional adaptations is essential for clinical assessment and rehabilitation planning, and provides a pathogenic basis for the following section.

### 4.5 Clinical quantification and multimodal assessment

**Figure 3:**
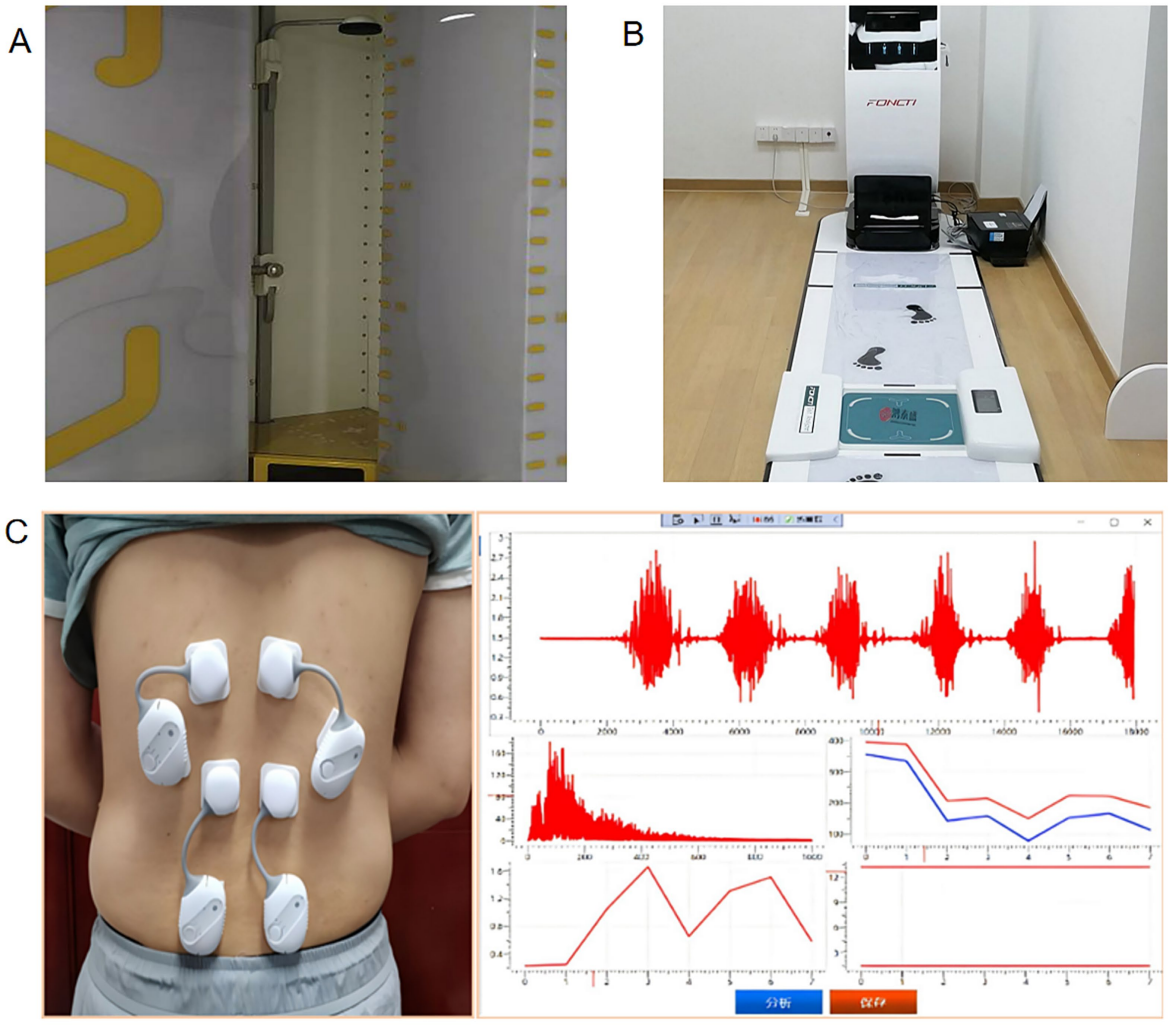
Pictures of professional equipment related to multimodal assessment **(A–C)**. EOS **(A)**. A gait analysis device that is recognized by a computer **(B)**. A surface electromyography device **(C)**.

The EOS system is based on the principle of planar sensing imaging, which can obtain three-dimensional sagittal images from the cranial base to the foot in a single acquisition, truly reflecting the overall alignment of the spine-pelvis-lower limb. Figure 3A shows the EOS-related detection equipment. Compared with supine CT, EOS can reveal the true compensatory posture under load, providing a unique perspective for the compensatory mechanisms of the spine-lower limb (51).

The EOS system provides multiple key parameters in the assessment of spinal-pelvic-lower limb alignment. As mentioned above, SVA is the most commonly used parameter for evaluating overall sagittal balance of the spine. The T1 Pelvic Angle (TPA) is defined as the angle formed by the line connecting the center of the T1 vertebral body and the center of the femoral head, and the line connecting the center of the femoral head and the midpoint of the upper endplate of the sacrum; it integrates information from both trunk inclination and pelvic rotation. In a cross-sectional study (Beyer et al.), when patients had a TPA ≥ 14°, their average ODI reached over 20 points (52). This may indicate a significant positive correlation between TPA and the quality of life of ASD patients. Besides, the Knee Flexion Angle (KFA) refers to the angle between the femur and tibia, while the Ankle Dorsiflexion Angle (ADA) is the angle formed between the tibia and the plantar surface in the sagittal plane. Both parameters together constitute important indicators for evaluating lower limb joint compensation (53). Table 3 lists the normal ranges and clinical significance of the main parameters in EOS evaluation. It must be noticed that when measurement values are outside the normal range but have not reached the abnormal threshold, it may indicates that the joint is in a transitional stage between injury and non-injury (Table 3).

**Table 3:** Important indicators for evaluating spinal imbalance compensation in EOS.

Parameter	Normal range	Abnormal threshold	Clinical significance
SVA (mm)	<50 mm	50 mm	Evaluating overall sagittal imbalance
TPA (°)	<10°	14°	Comprehensive assessment of spinal alignment and pelvic compensation
PT (°)	≤20°	20°	Reflecting the degree of posterior pelvic tilt
KFA (°)	<30°	30°	Adapting to imbalance caused by uneven ground or forward shift of the center of gravity
ADA (°)	<20°	30°	Maintaining the stability of the body’s center of gravity when standing and prevent falls.

Three-dimensional gait analysis characterizes dynamic compensatory patterns in patients with adult spinal deformity (ASD). Current gait assessment methods primarily utilize three approaches: optical motion capture, wearable sensors, and computer vision. Optical motion capture employs infrared cameras to track reflective markers, reconstructing 3D kinematics of the spine and joints. Wearable sensors, based on inertial measurement units attached to body segments, record real-time acceleration and angular velocity to derive joint angles throughout the gait cycle. Emerging computer-vision techniques analyze walking videos captured with conventional cameras, extracting motion data via deep-learning algorithms for marker-free gait analysis (54–56). Figure 3B shows a gait detection and analysis device based on computer machine vision. In a gait analysis based on a three-dimensional motion capture system (Huysmans et al.), patients with ASD exhibited significantly greater left–right swing amplitudes compared to the control group (sacrum: +14.0 mm; upper back: +23.3 mm), along with a decrease in walking speed by 0.04 m/s, a reduction in step length by 0.06 m, and an extension of double support phase by 0.02 s (57). This may suggest that patients with ASD have markedly reduced walking efficiency, decreased stability, and increased fall risk under compensatory conditions.

**Figure 4:**
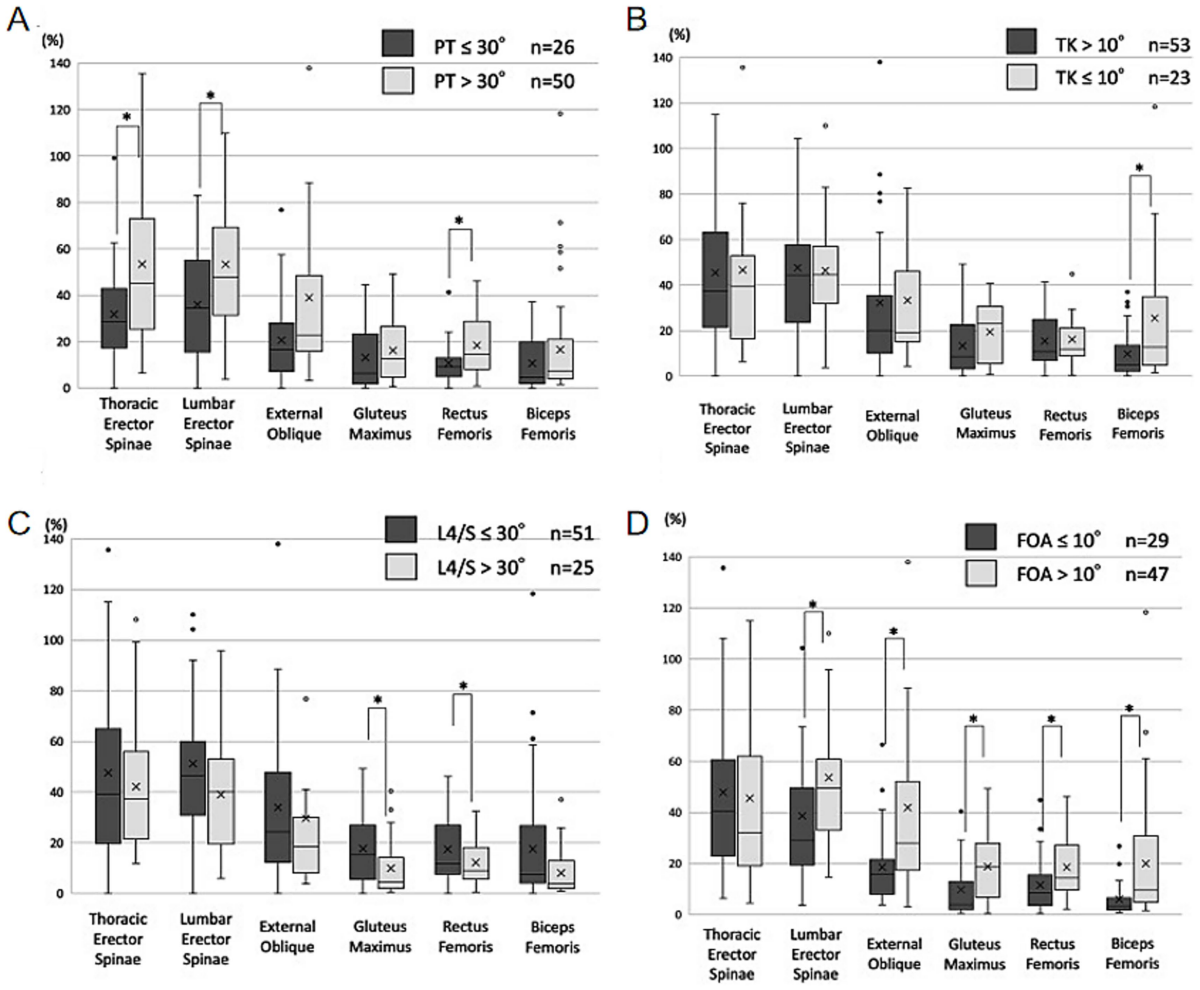
Muscle activity with and without compensation box-and-whisker diagram. **(A)** Pelvic retroversion. **(B)** Reduction of thoracic kyphosis. **(C)** Hyperextension of lumbosacral junction. **(D)** Knee flexion. Adapted with permission46. Copyright 2023, Measuring Muscle Activity in the Trunk, Pelvis, and Lower Limb Which Are Used to Maintain Standing Posture in Patients with Adult Spinal Deformity, With Focus on Muscles that Contract in the Compensatory Status.

The sEMG provides insight into neuromuscular control strategies in individuals with spinal sagittal imbalance. Figure 3C illustrates a surface electromyography device. Surface electromyography analysis (Yamato et al.) indicates that a PT > 30° may associated with increased activation of the TES (+20%), lumbar erector spinae (LES; +16%), and rectus femoris (RF; +7%) compared to controls. Similarly, knee flexion exceeding 10° led to elevated activation in multiple muscles, including the LES (+15%), abdominal external oblique (AEO; +23%), RF (+7%), BF (+14%), and GM (+9%). Differences in muscle recruitment between compensatory and non-compensatory states in ASD patients are summarized in Figure 4. These findings may suggest that sagittal imbalance triggers a compensatory mechanism reliant on multi-muscle synergy, which may contribute to elevated energetic demand during movement (46).

To achieve the integration of multimodal indicators, a three-dimensional evaluation framework centered on EOS (EOS imaging), gait analysis, and electromyography (EMG) can be established, coupling imaging data, kinematic data, and EMG indicators. Within the RCP evaluation framework: EOS provides the compensatory reserve of each joint under static conditions; gait analysis offers the functional compensation cost during dynamic movement; EMG analysis can be used to predict potential fatigue risks. Multimodal quantitative assessment provides an objective tool for integrated research on the spine-pelvis-lower limb system. In the future, combining artificial intelligence-based gait recognition with EOS three-dimensional modeling will enable precise prediction and individualized intervention for degenerative spinal deformities.

### 4.6 Clinical implications and comprehensive intervention strategies

Understanding and actively managing compensatory states is not only about improving posture but also about delaying degeneration and optimizing the outcomes of orthopedic surgery.

**Figure 5:**
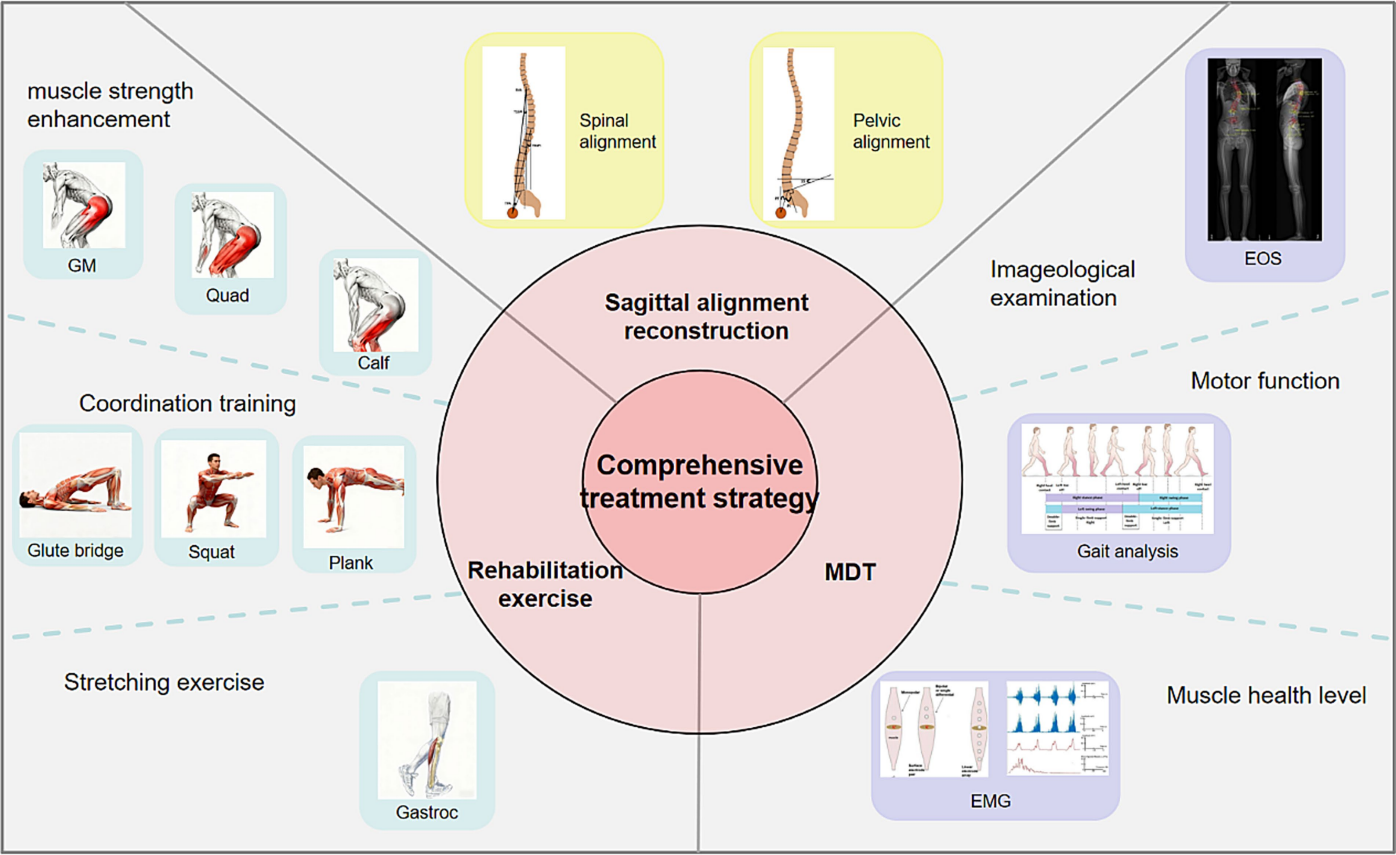
Schematic diagram of postoperative comprehensive health management model for adult spinal deformity patients.

For patients presenting with lower limb compensation, the treatment should evolve from merely “orthopedic reduction” to “holistic chain recovery”. The comprehensive management plan is structured around three core levels: ① Spinal–Pelvic Alignment Reconstruction: Precise spinal corrective surgery, combined with individualized matching of pelvic parameters, reestablishes a stable spinopelvic sequence. This addresses the root cause by reducing abnormal biomechanical loading on lower limb joints. ② Systematic Lower Limb Rehabilitation: Postoperative rehabilitation follows a three-dimensional framework of strength – coordination – flexibility. Strength training targets key extensor muscles within the sagittal plane kinetic chain, including the gluteus maximus, quadriceps femoris, and triceps surae. Coordination training employs closed-chain exercises (e.g., bridges, squats, balance-pad activities) to restore synergistic hip–knee–ankle movement patterns during weight-bearing, thereby improving gait and energy efficiency. Flexibility training involves systematic stretching of muscles prone to shortening due to chronic flexion contracture, such as the hamstrings and gastrocnemius, aiming to restore joint range of motion and minimize injury risk from passive overstretching. ③ Multidisciplinary Dynamic Monitoring and Management (MDT): Regular quantitative assessment of key radiographic and clinical parameters—including PT, KFA and ADA—enables dynamic evaluation of compensatory patterns. This guides scientifically based adjustments and progression of rehabilitation intensity, facilitating individualized, step-wise rehabilitation management. Figure 5 illustrates the integrated perioperative health management model for ASD patients (58, 59) (Figure 5).

This chapter reveals the mechanical logic of ‘deformation and compensation’, while the next chapter will further explore the ‘pathological consequences of compensation’—that is, how this abnormal stress state evolves into multi-joint degeneration and systemic diseases.

## 5 Pathological consequences of compensation and mechanism of degenerative evolution

### 5.1 General overview: from protective compensation to damaging compensation

**Figure 6:**
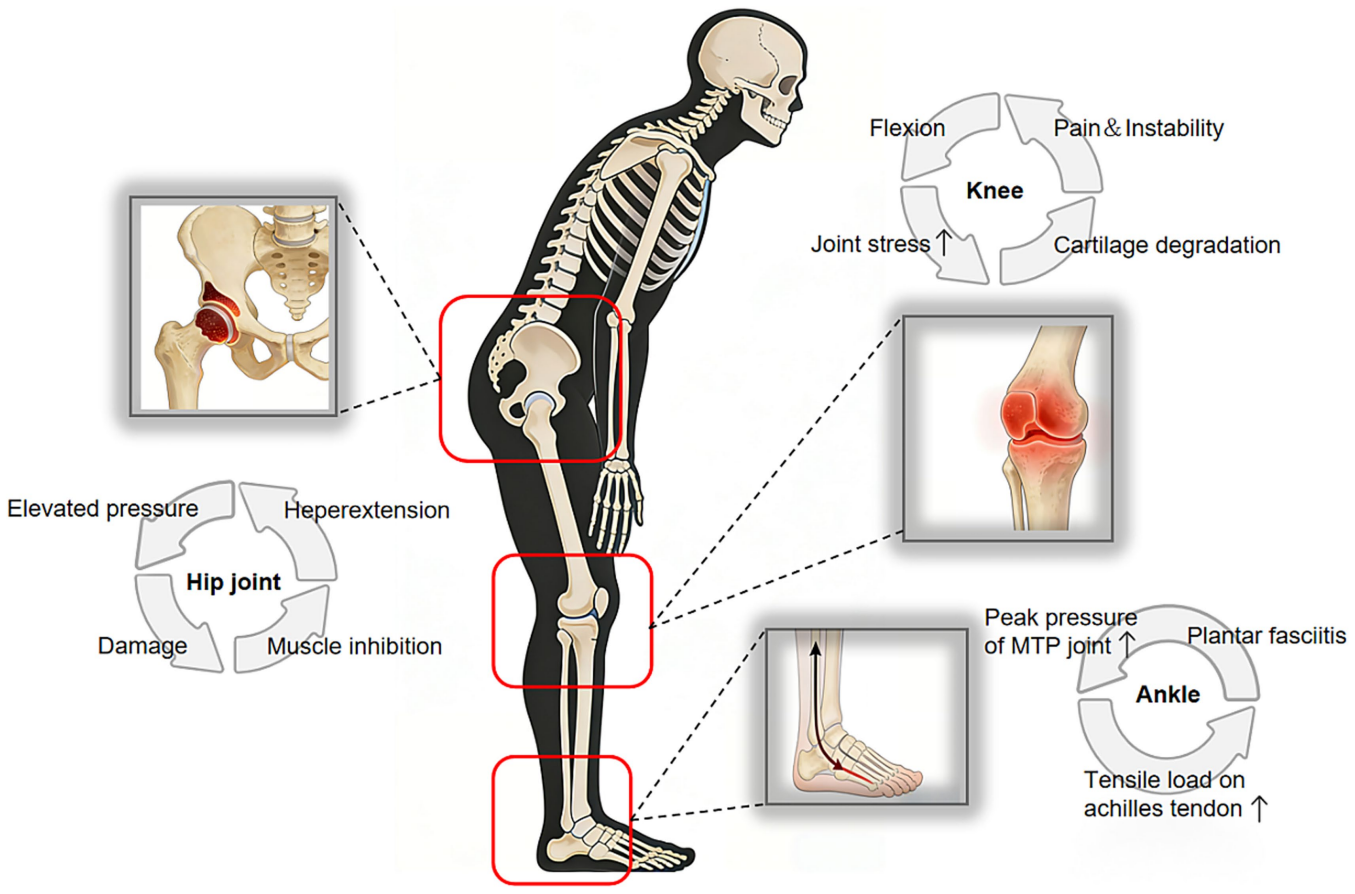
Schematic representation of pathological changes in the lower limb joints secondary to sagittal spinal imbalance.

A musculoskeletal modeling study demonstrated that patellofemoral joint pressure rises more than eightfold during full knee flexion, significantly increasing the risk of pain. This effect may be attributed to a fixed articular contact area and limited load-bearing capacity of periarticular tissues. Under compensatory conditions, abnormal mechanical distribution and transmission can generate stresses that exceed local tissue tolerance, resulting in micro-injury, inflammation, and structural changes (60). However, direct evidence for this mechanism remains scarce in patients with degenerative spinal deformity. Figure 6 outlines the overall pattern of pathological changes in lower limb joints during compensatory spinal imbalance (Figure 6). To further elucidate this progression, the following analysis will examine the hip, knee, and ankle joints sequentially.

### 5.2 Hip joint: degeneration of the anterior superior region induced by hyperextension compensation

The compensatory priority of pelvic posterior rotation and hip extension stems from their largest inherent sagittal-plane range of motion. Powered by the body’s strongest muscle groups, it can generate considerable torque for postural adjustment. Consequently, hip compensation tends to occur early and often remains clinically subtle due to its substantial compensatory capacity. The subsequent increase in hip loading and soft tissue tension forms a biomechanical basis for joint degeneration and soft tissue injury.

Specifically, pelvic and hip compensation shifts the femoral head contact area within the acetabulum from the central to the anterosuperior region, concentrating pressure on the anterosuperior cartilage quadrant. Finite element analyses indicate that in developmental dysplasia of the hip (DDH), pelvic retroversion increases by an average of 6°, with a corresponding rise in maximum joint contact pressure of 1.5 MPa (61). Furthermore, in hip models (normal, borderline DDH, and DDH), the highest von Mises stresses were observed at a PT of 20° (1.7 MPa, 3.5 MPa, and 5.1 MPa, respectively) (62). This is consistent with the critical values noted in Table 3. Although the changes in von Mises stress in patients with DDH cannot be entirely equivalent to those in healthy individuals, the trend of stress alteration and biomechanical principles they reflect are highly similar to the changes in hip joint loading during pelvic retroversion in ASD patients. This chronic abnormal stress concentration may induce chondrocyte microcracks and compromise the cartilage-bone interface integrity. Microcracks can act as pathways, facilitating vascular invasion into the subchondral bone and bidirectional diffusion of pro-inflammatory cytokines (e.g., IL-1β, TNF-*α*) and growth factors. This process initiates abnormal subchondral bone remodeling, characterized by accelerated bone turnover, trabecular thinning, and radiographically evident bone marrow edema. Concurrently, stress-stimulated chondrocytes and osteoclast precursors appear to release increased levels of matrix-degrading enzymes and osteoclast-stimulating factors, establishing a positive feedback loop that exacerbates cartilage matrix degradation and aberrant bone structural remodeling (63, 64).

Furthermore, these biomechanical changes may initiate a vicious cycle with soft tissue biological responses. Excessive hip extension can lead to elevated pressure and soft tissue injury in the anterosuperior region, resulting in pain and inflammation that may inhibit the function of periarticular stabilizing muscles. Weakened gluteal muscle control could then further compromise pelvic stability, promoting even greater reliance on hip hyperextension for compensation. This in turn exacerbates stress concentration anterosuperiorly, creating a self-perpetuating loop of hyperextension, elevated pressure, tissue injury, and muscular inhibition. In support of this mechanism, a cohort study indicates that reduced PT is associated with specific types of acetabular impingement (notably Types 1B and 2B), which are frequently linked to restricted posterior pelvic tilt and may predispose to anterosuperior impingement during hip flexion (65, 66). This suggests that even as the hip joint continues to serve a compensatory role, its ability to maintain mechanical stability may be compromised.

As pain related to hip joint injury intensifies, the activity of the hip extensor muscle group, primarily the gluteus maximus, is inhibited. When the hip joint struggles to independently maintain sagittal plane balance, compensatory load shifts distally to the knee joint. The corresponding pathological changes will be discussed in detail in the next section.

### 5.3 Knee joint: pathology of high shear stress caused by flexion compensation

Knee flexion provides a rapid and energetically efficient means of shifting the body’s center of gravity posteriorly, serving as the most readily available postural compensation strategy. In this process, the quadriceps and hamstrings coactivate to modulate the degree of flexion, enabling the knee to respond swiftly to postural adjustments. This compensatory mechanism for maintaining upright posture through knee flexion, however, imposes substantially elevated mechanical stress on both the patellofemoral and tibiofemoral joints.

Knee joint degeneration results from the combined effects of shear and compressive forces. With increasing knee flexion, the contact pressure in the patellofemoral joint (PFJ) rises more rapidly than the contact area (67). In a simulation of peak loads during adult lower-limb movement, PFJ pressure increased exponentially with flexion, reaching up to 8.2 times body weight during a single-leg squat (45). This suggests that sustained compression may predispose patellar cartilage to microfibrillation, fissuring, and softening—a potential pathological basis for anterior knee pain. Meanwhile, at the tibiofemoral joint, a substantial knee extension moment generated to counteract anterior shift of the center of gravity elevates anterior–posterior shear forces within the joint. Consequently, the load on the anterior cruciate ligament (ACL), which restrains anterior tibial translation, rises sharply. The tibial plateau cartilage, particularly in the medial compartment, is therefore exposed to peak shear stress, accelerating cartilage wear and degeneration (68). Additionally, structures such as the fat pad, synovium, and menisci, under high mechanical stress, undergo repeated compression and friction, leading to persistent low-grade inflammation and tissue injury. Under abnormal loading, mechanosensitive ion channels (e.g., PIEZO1) in articular cartilage are activated, triggering the NF-κB inflammatory pathway and upregulating degradative enzymes including MMPs and ADAMTS, which promote rapid breakdown of the extracellular matrix (69, 70).

These interlinked processes establish a positive feedback loop: initial compensatory flexion induces abnormal stress, which through mechanotransduction promotes inflammation and matrix degradation, leading to chondrocyte death. This results in structural cartilage damage, reduced load-bearing capacity, pain, and quadriceps inhibition. To mitigate further joint injury, the body adopts even greater flexion as compensation (71, 72), thereby trapping the joint in a self-perpetuating cycle of ‘flexion compensation—abnormal stress—cartilage degradation—pain and instability—further flexion compensation’. Consequently, pain and functional decline often manifest early in this compensatory cascade, even as the chain of compensatory reactions continues to propagate.

Sustained knee flexion compensation elevates energy expenditure and reduces gait efficiency. Concurrent pain-mediated quadriceps inhibition contributes to instability, often manifesting as knee buckling. As the compensatory capacity of the knee nears exhaustion, compensatory loading inevitably shifts distally to the ankle and foot. Although the ankle ultimately bears the terminal load in this chain, the site of pain transitions from the anterior knee to the ankle and foot—a progression that will be the focus of the following section.

### 5.4 Ankle and foot: terminal compensatory limit injury

The foot-ankle complex acts as a “terminal compensator” due to its distinct anatomical and functional role: it can modulate the magnitude and direction of ground reaction forces via subtle postural adaptations without substantially altering global body alignment (73). This end-stage compensatory strategy comes at the cost of marked load concentration and tissue overloading.

The pathological progression of ankle compensation manifests as a pattern of high-intensity focal loading, driven by a closed-loop mechanism of mechanical overload. Persistent excessive ankle dorsiflexion induces relative posterior tilt of the tibia, resulting in abnormal posterior-directed loads on the metatarsal heads. This leads to a focal zone of chondral contact that significantly elevates peak pressure within the metatarsophalangeal joints. Simultaneously, the Achilles tendon operates under high tension to counteract the forward torque of the body, which may disrupt collagen microarchitecture and tendon metabolism, thereby increasing the risk of degenerative tendinopathy. These alterations collectively shift plantar pressure anteriorly. Consequently, the plantar fascia is subjected to a triple overload: high tension from the Achilles tendon, repetitive impact during gait, and increased shear from anterior pressure redistribution. Microtrauma accumulates faster than the tissue’s repair capacity, accelerating the transition from physiological strain to plantar fasciitis. Given the concentration of stress at limited sites such as the calcaneal attachment and metatarsal heads, focal injury develops more rapidly than the chronic degeneration seen in larger joints like the hip or knee. This explains the swift distal migration of clinical symptoms from the trunk to the foot and ankle. Thus, once the ankle-foot complex becomes the terminal compensator, symptoms often escalate in an acute or subacute manner, rather than following a gradual progressive course (74, 75).

Talar cartilage lesions typically cause dorsiflexion pain and restricted ankle mobility, while Achilles tendinopathy presents with morning stiffness, weight-bearing pain, and impaired athletic performance. An anterior shift in the center of plantar pressure further elevates the risk of plantar fasciitis and metatarsal stress injuries. Collectively, these local pathologies lead to pain, muscle fatigue, and load aversion, triggering protective gait adaptations such as shortened stride length, reduced speed, and prolonged double-limb support. This stage indicates that the body’s compensatory system is approaching a decompensation threshold. Patients exhibit diminished coordinated control of the ankles, and the trunk fails to achieve sagittal-plane stability via the lower limbs, significantly increasing fall risk (76, 77). Thus, ankle and foot injuries are not merely local pathologies—they signal that lower-limb compensatory mechanisms are nearing physiological limits. When the physical cost of compensation exceeds its stabilizing benefits, the system transitions from a compensated to a decompensated state.

## 6 Multimodal assessment framework for RCP

### 6.1 Definition and clinical significance of RCP

In degenerative spinal deformity, overall balance is governed by the severity of spinal imbalance and compensatory mechanisms in the lower limbs. RCP refers to the capacity of the pelvis, hip, knee, and ankle joints to sustain global balance prior to decompensation in pathologies such as degenerative spinal deformity and lower limb joint degeneration. RCP reflects not only a patient’s biomechanical reserve in sagittal imbalance but also carries considerable clinical relevance. Quantifying RCP allows clinicians to evaluate the progression of postural imbalance toward high fall risk and to anticipate the restoration of gait stability following corrective spinal surgery.

Postural balance depends on the coordinated function of the spinal-pelvic-lower limbs alignment. After spinal degeneration, compensatory mechanisms—including pelvic retroversion, hip hyperextension, knee flexion, and ankle dorsiflexion—are recruited to temporarily preserve overall balance. With disease progression, balance cannot be maintained, presenting clinically as gait instability, joint pain, and functional decline. Assessing RCP helps determine whether such compensatory mechanisms remain effective, identifying the threshold of decompensation and enabling early intervention to limit further impairment. The magnitude of RCP is modulated by both intrinsic biological traits and extrinsic behavioral factors. Intrinsic determinants include age-related joint degeneration, as well as the duration and severity of spinal deformity, which together define the baseline compensatory capacity and its rate of depletion. Lifestyle and physical activity also significantly influence RCP: sustained regular exercise helps maintain joint mobility and muscle strength, thereby preserving compensatory potential, whereas sedentary behavior accelerates its decline.

**Figure 7:**
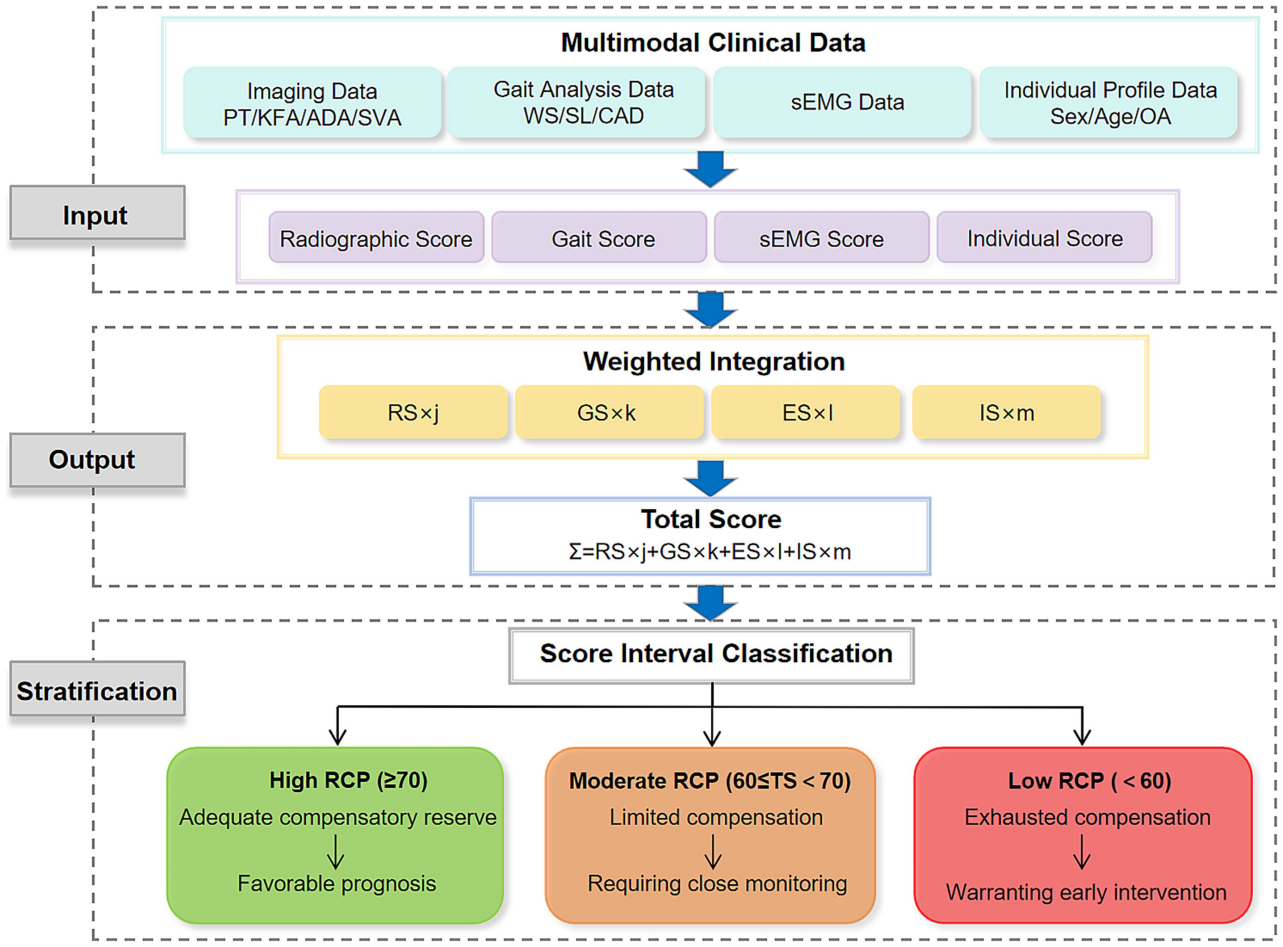
A workflow for quantitative assessment of RCP.

**Figure 8:**
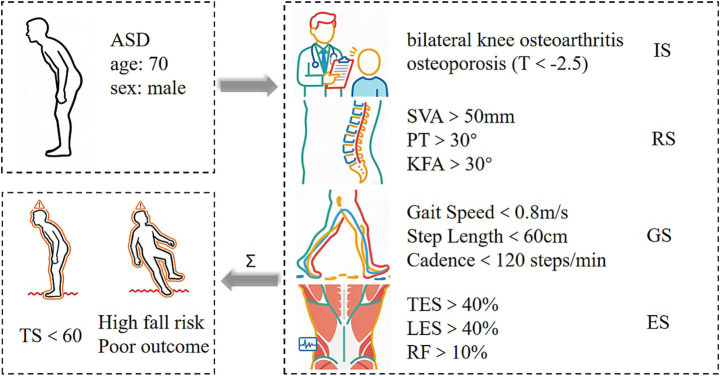
Example patient RCP calculation.

In clinical practice, the RCP can be quantified by integrating weighted scores from four domains: radiography, gait, EMG, and individual clinical history. Weighting should reflect both evidence-based correlations with key outcomes (e.g., functional decline, fall risk, surgical recovery) and expert consensus. A standardized workflow for calculating the composite RCP score is presented in Figure 7 (Figure 7). As an illustrative example, consider a 70-year-old male with a 10-year history of ASD and bilateral knee osteoarthritis (Figure 8). Following the RCP assessment protocol: (1) Data collection: EOS imaging provided sagittal parameters (SVA, PT, KFA, ADA); 3D gait analysis captured speed and stability metrics; surface EMG recorded paraspinal muscle activity; and clinical history included age and osteoarthritis status. (2) Domain-specific scoring: Each domain was converted to a normalized score (Radiographic Score, Gait Score, EMG Score, Individual Score). (3) Weighted integration: The composite RCP score was computed as *Σ* = (RS × j) + (GS × k) + (ES × l) + (IS × m), where j, k, l, m denote evidence-based weights. (4) Stratification: The resulting score places the patient within a prognostic category defined by future large-scale cohort studies, thereby offering actionable guidance for clinical management. This approach transforms multimodal data into a single, interpretable metric that supports individualized decision-making in complex spinal deformity. This paper presents a visualized, implementable preliminary framework, though its specific parameters and scoring methods require further research.

### 6.2 Multimodal quantitative Indicator system of RCP

Human compensatory mechanisms involve coordinated actions across multiple joints and muscle systems, making a single metric insufficient to fully capture compensatory capacity. Therefore, multimodal assessment is often necessary to evaluate compensatory potential comprehensively. In patients with ASD, the most commonly used quantitative tools include imaging parameters, gait analysis, and electromyography. This integrated approach overcomes the limitations of any single modality by combining complementary indicators, thereby improving the overall validity and accuracy of the assessment. It offers a reliable basis for clinically evaluating compensatory status and guiding intervention strategies.

To facilitate the clinical translation of multimodal assessment, this study proposes a hierarchical and scalable integration framework, comprising core and extended protocols, along with clear guidelines for handling missing data. The core protocol is designed for broad clinical implementation, prioritizing accessibility and efficiency. It includes: (1) standing whole-spine radiography to quantify key sagittal parameters (SVA, PT, TPA, KFA) for evaluating spinopelvic alignment and compensatory reserve; (2) simplified gait assessment using gait speed, step length, and either the Timed Up and Go (TUG) or 6-Minute Walk Test (6MWT) to reflect walking function and endurance; (3) if sEMG is unavailable, manual muscle testing or validated fatigue scales (e.g., Borg scale) should be used to approximate muscle function. This set focuses on fundamental imaging and functional metrics relevant to stablity and compensatory capacity. It is worth emphasizing that this core protocol is feasible even in resource-limited settings hospitals. The extended protocol is intended for tertiary hospitals or research settings, enhancing the core protocol with advanced measures: (1) EOS-based whole-body coronal and sagittal alignment analysis; (2) 3D motion capture or inertial sensor-based gait analysis with energy cost assessment; and (3) surface EMG to quantify neuromuscular parameters such as co-contraction index and muscle fatigue. This tier enables a more detailed, dynamic profiling of compensatory mechanisms. To address data gaps, a hierarchical substitution principle is applied: functional tests replace missing EMG data; simplified gait metrics substitute for absent 3D gait analysis. Critically, every assessment must include radiographic data and at least one functional module. This structured approach ensures that essential compensatory indicators are obtainable across varied clinical resources while supporting comprehensive research-grade evaluation, thereby improving the practicality and standardization of multimodal assessment. Future efforts to standardize protocols and refine techniques can enhance the clinical utility and reliability of RCP assessment (78–80).

### 6.3 Relevant threshold judgment for RCP

In RCP assessment, a threshold represents a clinically defined cut-off value based on specific quantitative indicators. When multiple integrated indicators exceed or fall below this threshold, it suggests that the patients’ compensatory reserve is nearing depletion, indicating a risk of accelerated functional decline. Establishing such thresholds within a multimodal framework enables clinicians to more accurately evaluate remaining compensatory capacity and guide decisions regarding surgical or other interventions. Different thresholds apply to distinct clinical scenarios, such as preoperative evaluation and postoperative monitoring. In this study, the ‘threshold’ is conceptualized not as a single cut-off but as a stratified system comprising distinct boundaries and transitional zones. Hard cutoffs are delineated values supported by high-level evidence or consensus (e.g., SRS-Schwab classification: PT > 20° denotes abnormality; PT > 30° indicates severe decompensation). Exceeding these values signifies a parameter has entered a pathological or decompensated state. Soft zones represent intervals just below these hard cutoffs (e.g., PT 15°–20°). Values within a soft zone deviate from the ideal, signaling that the compensatory system is under stress, though still operative. Therefore, within our multimodal assessment framework—where parameters like TPA and ADA provide complementary information—we propose employing soft zone criteria. Final classification should require multi-indicator consistency, defined as ≥2 parameters concurrently exceeding their respective soft zone thresholds. This approach enhances sensitivity for detecting early compensatory failure while maintaining diagnostic specificity.

Furthermore, the RCP threshold is not static. Implementing a stratified threshold strategy based on individual characteristics (e.g., age, sex, prior joint pathology, bone health status) can improve assessment accuracy. Notably, osteoporosis undermines the structural foundation of the compensatory chain by weakening vertebral bone and accelerating compression fractures, thereby reducing physiologic reserve. Consequently, in patients with severe osteoporosis—even those who are younger or lack significant joint degeneration—functional compensatory capacity is diminished, and threshold assessment should adopt more conservative criteria, aligning with those used for older adults. For instance, in elderly patients (with 65 years suggested as a preliminary reference cut-off) with diminished physiological compensation and a higher prevalence of degenerative disease, lower diagnostic thresholds enhance sensitivity for early risk detection. Conversely, younger or highly active individuals, who typically exhibit greater physiological reserve and functional adaptation, may be evaluated using relatively higher thresholds to minimize over-intervention (81–83). It should be emphasized that this chronological age cut-off requires integration with the patient’s “physiological age”—for example, a physically active 65-year-old may be assessed using younger-group thresholds, while a 55-year-old with significant comorbidities (such as severe osteoporosis or advanced osteoarthritis) should align with older-group thresholds. This tailored approach maintains diagnostic specificity while improving the detection of incipient decompensation, thereby supporting more personalized and proactive management of spinal deformity. Table 4 presents an example of threshold adjustment methods used for different patient statuses (Table 4).

**Table 4:** A suggested adjustments to spinopelvic parameter thresholds based on clinical scenarios.

Clinical scenario	Recommended thresholds			
	TPA (°)	PT (°)	KFA (°)	ADA (°)
Young age/Slight imbalance (81)	14	25	35	30
Advanced age/Marked imbalance (81)	10	15	25	20
Hip/Knee OA: Present (83, 85)	12	15	25	25

### 6.4 Association of RCP with surgical outcomes/prognostic function

In the management of spinal sagittal imbalance, radiographic correction does not consistently translate into functional improvement. Therefore, surgical outcomes should be evaluated not only by alignment parameters but also by indicators that reflect the efficacy of converting morphological correction into functional benefit (84). Quantifying RCP may help assess both the risk of imbalance progressing to falls and the potential for gait recovery after surgery. As a potential link between preoperative compensation and postoperative functional outcomes, RCP could also provide a rationale for preoperative stratification, threshold determination, and postoperative rebound risk.

RCP may serve as a clinical variable for preoperative risk stratification and outcome prediction. Its core role is to quantify the residual capacity of the compensatory chain and suggest whether realignment after surgery might translate into stable gait and functional gain. Consistent abnormalities across multiple indicators can more reliably identify exhausted compensatory potential, thereby informing surgical decision-making and providing a basis for postoperative functional monitoring.

Patients with low RCP may indicate high risk of postoperative posture recovery difficulty and imbalance rebound due to degenerative and stiffened joints. In contrast, those with high RCP may be associated with pain relief and improved ambulation. Whether spinal realignment translates into stable gait and functional improvement may depend on the remaining availability of the lower-limb compensatory chain. When compensatory capacity nears exhaustion—manifested as gait instability, joint pain, and functional decline—the body requires postoperative “reallocation of load line and re-establishment of neuromuscular control” yet lacks adequate biomechanical reserve, which could increase rebound risk. Since a key outcome difference lies in dynamic stability and compensatory release, postoperative assessment should extend beyond static radiologic parameters (e.g., SVA, PT/TPA) to include gait and muscle loading changes, thereby dynamically evaluating whether correction yields functional benefits. Establishing a multimodal assessment framework centered on EOS (whole-body weight-bearing alignment), 3D gait analysis (kinematic/spatiotemporal parameters), and sEMG (neuromuscular compensatory load) can integrate radiographic, kinematic, and electrophysiological measures to objectively quantify improvement in compensatory patterns and restoration of gait stability.

To validate the clinical relevance of this multimodal assessment framework, future studies could incorporate endpoints such as patient-reported outcomes (e.g., ODI scores), improvement in functional gait parameters, fall incidence, reoperation rates, and dynamic changes in long-term quality of life. These endpoints would help objectively examine the association between RCP-based assessment and postoperative functional recovery, gait stability, and overall surgical success.

## 7 Discussion and outlook

In traditional management of spinal deformity, correction of radiographic parameters has been the central goal, based on the premise that achieving alignment targets leads to functional improvement. However, even when alignment standards are met, outcomes vary widely in terms of pain relief, gait recovery, energy efficiency, and risk of recurrent imbalance. This variation depends not only on spinal correction, but more on whether the pelvis–lower limb compensatory chain retains sufficient capacity for physiological reset after prolonged overuse in a decompensated state. We propose a shift toward a chain-compensation paradigm, which reframes the treatment objective from merely correcting angles to restoring coordinated mechanical function across the spine–pelvis–lower limb system. This approach integrates morphological correction with long-term functional recovery. The key to implementing this paradigm is the residual compensatory potential, a quantifiable measure that allows objective, stratified assessment of compensatory capacity beyond empirical judgment. Thus, treatment goals should expand beyond radiographic targets to include reconstruction of the compensatory chain, improved gait stability and energy efficiency, and reduced risk of re-imbalance, making functional recovery an endpoint equal in importance to alignment correction.

The clinical translation of chain-compensation thinking into improved outcomes requires its implementation as an integrated, closed-loop pathway spanning assessment, decision-making, surgery, and rehabilitation. Preoperatively, risk stratification and functional prognosis should utilize compensatory-chain indicators such as RCP. The focus is on identifying patients with nearly exhausted compensatory reserves, aiming to reduce baseline risks of persistent trunk anterior tilt and long-term recurrent imbalance. Intraoperatively, correction strategies must extend beyond restoring spinal sagittal parameters to achieve functional realignment of the global sagittal axis. Postoperatively, three-dimensional rehabilitation—emphasizing muscle strength, coordination, and flexibility—facilitates the shift from a passive compensatory state to long-term stability. The key endpoints are restoring or enhancing residual compensatory capacity, improving gait efficiency, and delaying secondary joint degeneration. Collectively, it is crucial to implement preoperative stratification, intraoperative reconstruction, and postoperative rehabilitation, while using the same set of chained compensation index system for follow-up evaluation and calibration of intervention intensity. The ultimately formed sustainable closed loop of ‘evaluation-intervention-re-evaluation’ is key to translating the radiological success of deformity correction into long-term functional benefits for patients.

This review is the first to synthesize evidence linking the spinal-pelvic-lower limb compensatory chain to secondary joint degeneration, integrating previously disparate imaging, gait, and degenerative findings into a unified causal framework. This model shifts clinical focus from static radiographic alignment to dynamic stability and decompensation. It also establishes a common foundation for preoperative risk stratification, intraoperative planning, and postoperative rehabilitation. Within this framework, RCP serves not as an isolated parameter but as a quantitative hub, connecting imaging, gait, and neuromuscular control metrics. It is therefore positioned as a central input for future predictive modeling and individualized treatment planning. Future research should advance in three key directions Firstly, mechanistically, it is necessary to clarify the ‘stress-inflammation-metabolism’ pathway involved in the degeneration of articular cartilage and intervertebral discs, thereby completing the key component of ‘mechanically driven degeneration’. Secondly, technologically, developing AI-driven models that integrate multimodal data (e.g., imaging, 3D gait analysis, sEMG) is needed to predict postoperative function, re-imbalance risk, and rehabilitation response. Thirdly, clinically, prospective, stratification-based trials are required to evaluate the efficacy of RCP-targeted rehabilitation protocols in improving gait efficiency, slowing degenerative progression, and enhancing long-term quality of life. The ultimate goal is to anchor clinical practice in RCP, translating chain-compensation theory into an integrated pathway of standardized assessment, personalized surgery, prescriptive rehabilitation, and long-term follow-up. This shifts the endpoint of sagittal deformity treatment from radiographic correction to sustained functional improvement and delayed degeneration.

## 8 Limitations and future research agenda

This review has several limitations. First, our analysis primarily focused on sagittal plane imbalance, which is most closely linked to clinical symptoms and compensatory mechanisms. The potential impact of coronal malalignment on lower limb joint degeneration was not systematically addressed, representing an important direction for future investigation. Second, current epidemiological evidence directly comparing the prevalence of hip, knee, or ankle osteoarthritis between ASD patients and age-matched controls remains sparse; most available data are derived from single-center cohorts or indirect inferences. Large-scale, population-based studies are needed to establish definitive associations. Third, due to the paucity of finite element modeling studies based on asymptomatic individuals, some biomechanical inferences in this review—particularly regarding joint contact stress and tissue loading—were drawn from studies involving specialized populations (e.g., patients with DDH). Although these provide valuable mechanistic insights, they should be interpreted with caution when extrapolating to the general ASD population. Fourth, while modeling studies provide mechanistic insight (Level III), clinical correlation in large geriatric cohorts remains limited.

Therefore, we propose RCP as a novel and quantifiable metric. Pending further validation, it offers a practical starting point for personalized assessment. The most pressing need is to establish the external validity of the RCP framework through large-scale, prospective, multicenter cohort studies. Ultimately, the field must advance toward interventional research—particularly randomized controlled trials (RCTs)—to evaluate whether RCP-guided surgical and rehabilitative strategies yield superior long-term functional recovery and lower rebalancing rates compared with standard alignment-focused care.
